# Fermentation dynamics of yerba-mate and coffee-husk kombuchas: bioactive compounds, organic acids, volatile metabolites, and microbial succession

**DOI:** 10.3389/fnut.2026.1935769

**Published:** 2026-09-03

**Authors:** Luis Daniel Goyzueta-Mamani, Flavia Yuri Shimizu, Agnes de Freitas Diniz de Souza, Gilberto Vinicius Melo Pereira, Julia Dimbarre, Carlos Ricardo Soccol, Cristine Rodrigues

**Affiliations:** 1 Computational Biology and Chemistry Research Group, Vicerrectorado de Investigación, Universidad Católica de Santa María, Arequipa, Peru; 2 Department of Bioprocess Engineering and Biotechnology, Federal University of Paraná, Polytechnic Center, Curitiba, Paraná, Brazil

**Keywords:** amplicon sequencing, antioxidant capacity, coffee by-products, *Ilex paraguariensis*, kombucha, microbial succession, volatile profile

## Abstract

**Introduction:**

Although yerba-mate and coffee-husk have individually been explored as alternative kombucha substrates, it remains unclear how these chemically distinct matrices differentially shape metabolite evolution and microbial succession when fermented under equivalent conditions. This study directly compared yerba-mate kombucha (YMK) and coffee-husk kombucha (CHK) to investigate substrate-dependent chemical and microbial fermentation trajectories.

**Methods:**

YMK and CHK were produced using the same parental SCOBY and fermentation protocol. Infusions containing 10 g/L substrate and 60 g/L sucrose were fermented statically at 30 °C for 9 days and subsequently stored at 4 °C for 4 days. Phenolic and flavonoid contents, antioxidant responses, organic-acid profiles, volatile compounds, and bacterial and fungal community succession were evaluated.

**Results:**

The two matrices generated distinct fermentation trajectories. YMK maintained consistently higher phenolic content and antioxidant-related responses, reaching 467.97 ± 43.09 μg GAE/mL at the end of refrigerated storage, and exhibited a broader and more persistent distribution of volatile chemical classes. CHK showed lower but comparatively stable antioxidant-related measurements and a more restricted volatile profile. Organic acids varied non-monotonically in both formulations, although YMK showed a higher final malic acid concentration. Komagataeibacter remained dominant throughout YMK fermentation, whereas CHK underwent a stronger temporal shift toward Acetobacter. Fungal communities were comparatively stable in both substrates and were dominated by *Starmerella* and *Dekkera*.

**Discussion:**

These findings demonstrate that substrate composition influenced both beverage chemistry and the temporal organization of the kombucha microbiota. By directly comparing a phytochemical-rich botanical infusion and an agro-industrial coffee by-product under standardized fermentation conditions, this study extends previous single-substrate investigations and highlights the potential of coffee-husk as a plant-derived ingredient for sustainable fermented beverage development.

## 1 Introduction

Kombucha is a fermented beverage produced through the activity of a symbiotic culture of bacteria and yeasts (SCOBY) in a sweetened plant infusion. During fermentation, yeasts hydrolyze sucrose and produce ethanol, carbon dioxide, higher alcohols, and other intermediates, while acetic acid bacteria oxidize ethanol and aldehydes to organic acids and contribute to cellulose-pellicle formation. These transformations modify the chemical and sensory characteristics of the original infusion. However, the composition of the final beverage is not determined by the inoculum alone; it also depends strongly on the botanical matrix, fermentation conditions, and interactions between substrate-derived compounds and the microbial consortium (1–3).

The plant substrate supplies fermentable nutrients, minerals, phenolic compounds, organic acids, and aroma precursors that can influence microbial growth and metabolite formation. Substrate composition, sucrose concentration, oxygen availability, temperature, inoculum structure, and fermentation time can therefore alter both microbial succession and the accumulation or transformation of organic acids, phenolic compounds, and volatile metabolites (4). Consequently, fermentation patterns obtained with conventional black or green tea cannot necessarily be extrapolated to chemically or structurally different botanical materials.

This substrate dependence has stimulated increasing interest in kombucha analogues produced from medicinal plants, regional infusions, fruit-derived materials, and agro-industrial by-products. Such substrates offer opportunities not only to diversify beverage composition but also to valorize plant residues through microbial processing. Nevertheless, the suitability of an alternative material cannot be inferred solely from its initial concentration of bioactive compounds. Its soluble nutrient fraction, structural complexity, accessibility to microbial enzymes, content of potential inhibitory compounds, and capacity to support the core kombucha community may also determine the resulting fermentation trajectory (5, 6).

Yerba-mate (*Ilex paraguariensis*) and coffee-husk represent two particularly relevant but contrasting matrices. Yerba-mate is a widely consumed South American infusion whose readily extractable fraction contains caffeine, chlorogenic and caffeoylquinic acids, flavonoids, minerals, and aroma precursors. Its soluble phytochemical composition may provide substrates for microbial biotransformation while maintaining a chemically rich beverage matrix (7, 8). Coffee-husk, in contrast, is an abundant agro-industrial by-product composed of residual carbohydrates, lignocellulosic material, caffeine, tannins, chlorogenic acids, minerals, and volatile precursors. Its use in fermentation may contribute to residue valorization, but its greater structural complexity and the presence of poorly extractable or potentially inhibitory constituents may produce a microbial and metabolic response distinct from that observed in a soluble botanical infusion (9–11).

Although both substrates have been individually explored as alternative matrices for kombucha production, they represent fundamentally different botanical systems. Yerba-mate is a phytochemical-rich leaf infusion traditionally consumed as a beverage, whereas coffee-husk is a lignocellulosic agro-industrial by-product with considerable potential for food valorization. Comparing these contrasting matrices under identical fermentation conditions provides an opportunity to evaluate how substrate composition influences microbial succession, metabolite production, and antioxidant-related characteristics while exploring sustainable strategies for coffee-processing residue valorization within a circular bioeconomy framework (4, 10, 11).

Fermentation may alter these matrices through the release, transformation, preservation, or degradation of native compounds and through the production of microbial metabolites such as organic acids, alcohols, aldehydes, and esters. At the same time, differences in nutrient accessibility may select different proportions of acetic acid bacteria and fermentative yeasts. These processes may generate products with different antioxidant-related chemical profiles and metabolite compositions. However, changes in microbial relative abundance or metabolite detection alone do not demonstrate increased probiotic, postbiotic, or host-related biological activity. Such properties require dedicated viability, functional, and biological assays that were beyond the scope of the present study.

Previous research has already demonstrated the feasibility of producing kombucha from yerba-mate and coffee-derived by-products. Yerba-mate kombucha has been investigated in relation to physicochemical and sensory properties and as a fermentation medium for bacterial cellulose and film production (7, 12). Coffee-husk or cascara kombuchas have likewise been evaluated for phenolic composition, antioxidant activity, acidity, sensory characteristics, and selected biological effects (13). These studies established the technological potential of each substrate individually but did not directly determine how yerba-mate and coffee-husk differ when fermented under the same standardized conditions and evaluated through a common, time-resolved analytical framework.

The novelty of the present study therefore lies not simply in using alternative kombucha substrates, but in directly comparing two chemically and structurally distinct plant matrices using the same parental SCOBY, substrate concentration, sucrose concentration, temperature, and fermentation period. Yerba-mate and coffee-husk kombuchas were evaluated through an integrated analysis of phenolic and flavonoid contents, antioxidant responses, quantitative organic-acid profiles, qualitative volatile-compound dynamics, and bacterial and fungal community succession. This design allowed us to investigate how substrate composition shaped coordinated chemical and microbial trajectories during fermentation and subsequent refrigerated storage.

## 2 Materials and methods

### 2.1 Kombucha fermentation

Yerba-mate (*Ilex paraguariensis*) was obtained from the commercial brand Mate Leão (Curitiba, Brazil). Coffee husks were obtained from dried husks of *Coffea arabica* beans produced at the Fortune Mountain Specialty Coffee farm (Teupasenti, El Paraíso, Honduras). Before infusion, the dried husks were ground to facilitate extraction.

Yerba-mate and coffee-husk infusions were prepared independently using 10 g/L plant material [1% (w/v)] and 60 g/L sucrose [6% (w/v)]. These concentrations were selected to provide an equivalent substrate loading and fermentable-carbon supply for both botanical matrices and are within the ranges commonly used for conventional and alternative kombucha fermentation (2, 4). For each biological replicate, 400 mL of infusion was prepared in a sterile 550-mL glass vessel. The plant material was infused at 80 °C for 10 min, filtered through qualitative filter paper, and cooled to room temperature. After cooling, each infusion was inoculated with previously fermented kombucha liquid at 10% (v/v), obtained from the same parental SCOBY culture. A cellulose pellicle approximately 0.8 cm thick, cut to match the surface area of the fermentation vessel (approximately one-third of the vessel surface), was added to each fermentation vessel. Three independent biological fermentations were prepared separately under identical experimental conditions and incubated statically at 30 °C for 9 days. These conditions were selected based on commonly reported kombucha fermentation protocols and applied uniformly to both substrates to enable a standardized comparison (2, 4). The vessels were covered with sterile breathable cloth to permit gas exchange and prevent external contamination. At day 9, the fermented liquids were transferred to sterile bottles and stored at 4 °C for an additional 4 days. This refrigerated period was considered a storage stage rather than an active second fermentation.

### 2.2 Sampling

Samples were collected at T0 (day 0), T1 (day 3), T2 (day 7), T3 (day 9), and T4 (day 13, after 4 days of refrigerated storage). At each sampling point, an aliquot of 10 mL was aseptically collected from each independent biological fermentation. Samples used for TPC, ABTS, and DPPH analyses were analyzed without prior centrifugation. For TFC determination, samples were centrifuged at 4,000 rpm for 3 min after completion of the colorimetric reaction, according to the analytical protocol. Samples were diluted with ultrapure water when necessary to maintain absorbance readings within the respective calibration ranges. Samples intended for chromatographic analyses were stored at 4 °C until analysis. Microbial community analyses were limited to T0–T3. Volatile-compound data were available for YMK from T0 to T4 and for CHK from T0 to T3.

### 2.3 Organic acids analysis

Organic acids were quantified using an Agilent HPLC system equipped with a refractive-index detector and a Hi-Plex H column (300 × 7.7 mm; Bio-Rad, Richmond, CA, United States). Separation was performed under isocratic conditions using 5.0 mM H_2_SO_4_ at a flow rate of 0.6 mL/min for 30 min. The column and detector temperatures were maintained at 60 and 50 °C, respectively, and the injection volume was 10 μL. Calibration curves were prepared for acetic, citric, malic, butyric, isovaleric, and lactic acids over the concentration range of 0.1–1.0 g/L, corresponding to 0.1–1.0 mg/mL. Analytes were identified by comparison with the retention times of authentic standards, and concentrations were calculated using their respective calibration curves.

### 2.4 Volatile compound analysis by GC–MS

Volatile compounds were analyzed by headspace gas chromatography coupled with mass spectrometry (HS-GC–MS). Briefly, 3 mL of each sample was transferred to a 10 mL sealed headspace vial and equilibrated at 60 °C for 30 min with agitation. Volatiles were introduced into the chromatographic system by static headspace injection. GC–MS analysis was performed to characterize temporal changes in the volatile and semi-volatile profiles rather than aroma perception or sensory-active compounds. Samples were analyzed from T0 to T4 for YMK and from T0 to T3 for CHK because the corresponding CHK-T4 sample was unavailable for GC–MS analysis.

Chromatographic separation was performed using an SH-Rtx-5MS capillary column (30 m × 0.25 mm internal diameter × 0.25 μm film thickness). Helium was used as the carrier gas at 1.0 mL/min. The injector temperature was 260 °C and the split ratio was 1:20. The mass spectrometer was operated in electron-ionization mode at 70 eV, and mass spectra were acquired over an m/z range of 30–250.

Compounds were tentatively identified by comparison of their mass spectra with the NIST 14 library using GCMSsolution version 2.2. Results were expressed as presence–absence data. Putative contaminants and artifact-like signals, including plasticizer- and silicon-related compounds, were retained in Table 1 for transparency but excluded from the biological interpretation and from the chemical-class summaries.

**Table 1:** Representative volatile and semi-volatile compounds associated with substrate differentiation, temporal persistence, and analytical interpretation.

Compound	Chemical class	YMK pattern	CHK pattern	Proposed relevance	Interpretation status
Linalool	Terpene	Persistent	Persistent	Floral/citrus aroma	Plausible matrix marker
α-Terpineol	Terpenoid alcohol	Recurrent	Sporadic	Floral aroma	Plausible
Eugenol	Phenol	Recurrent	Not detected	Spicy/phenolic note	YMK-associated
Megastigmatrienone	Norisoprenoid	Recurrent	Not detected	Tea-like aroma	YMK-associated
Phenylethyl alcohol	Aromatic alcohol	Persistent	Persistent	Floral/rose aroma	Shared marker
Nonanal	Aldehyde	Persistent	Persistent	Citrus/fatty note	Shared marker
Decanal	Aldehyde	Persistent	Recurrent	Citrus/waxy note	Shared marker
Furfural	Aldehyde	Early detection	Early detection	Processing-associated note	Tentative origin
Hexanal	Aldehyde	Early/transient	Intermittent	Green/grassy note	Temporal marker
Butanoic acid, 3-methyl-	Organic acid	Persistent	Persistent	Fermentation-associated aroma	Shared marker
Caffeine	Alkaloid	Persistent	Persistent	Matrix-associated compound	Qualitative detection
2,4-Di-tert-butylphenol	Phenolic signal	Persistent	Persistent	Possible bioactive or contaminant	Ambiguous origin; requires confirmation
Diethyl phthalate	Plasticizer-like ester	Detected	Not consistently detected	Possible contamination	Probable analytical contaminant; excluded from biological interpretation
Silanediol, dimethyl-	Silicon-related signal	Detected	Detected	Possible column/material artifact	Probable column/material-related artifact; excluded
Homosalate	Salicylate-like compound	Sporadic	Not detected	Possible contamination	Possible external contaminant; excluded

### 2.5 Total phenolic content

Total phenolic content was determined using the Folin–Ciocalteu method adapted from Singleton et al. (14). The Folin–Ciocalteu reagent was diluted 1:10 (v/v) in ultrapure water, and sodium carbonate was prepared at 7.5% (w/v). A gallic acid calibration curve was prepared over the concentration range of 4.128–113.52 μg/mL. Samples were analyzed without prior centrifugation. After incubation for 60 min at room temperature, absorbance was measured at 740 nm using a PowerWave XS microplate spectrophotometer (BioTek Instruments, Inc., Winooski, VT, United States). Results were expressed as μg gallic acid equivalents per mL of beverage (μg GAE/mL).

### 2.6 Total flavonoid content

Total flavonoid content was determined using the aluminum-chloride colorimetric method adapted from Zhishen et al. (15). Sodium nitrite, aluminum chloride, and sodium hydroxide solutions were prepared at 5% (w/v), 10% (w/v), and 1 M, respectively. A rutin calibration curve was prepared over the concentration range of 0.1–1.0 mM. After completion of the colorimetric reaction, samples were centrifuged at 4,000 rpm for 3 min, and absorbance was measured at 510 nm using a PowerWave XS microplate spectrophotometer (BioTek Instruments, Inc., Winooski, VT, United States). Results were expressed as μg rutin equivalents per mL of beverage (μg RE/mL). The rutin-equivalent concentrations obtained from the calibration curve were converted to μg RE/mL using the molecular mass of the rutin standard employed in the assay.

### 2.7 DPPH radical scavenging activity

DPPH radical-scavenging activity was determined using a microplate method adapted from Polez et al. (16). A 0.06 mM DPPH solution was prepared in analytical-grade methanol. The reaction mixture contained 150 μL of diluted sample and 450 μL of DPPH solution and was incubated for 30 min in the dark at room temperature. Absorbance was measured at 492 nm using a PowerWave XS microplate spectrophotometer (BioTek Instruments, Inc., Winooski, VT, United States). Appropriate sample blanks were included to correct for intrinsic sample color. DPPH inhibition was calculated as:


$$\begin{array}{l} \text{DPPH inhibition}\;(\%)=(A_{\text{control}}-(A_{\text{sample}}-A_{\text{blank}}))\\ /A_{\text{control}}\times 100 \end{array}$$

DPPH results were expressed as percentage radical inhibition because the adopted assay did not use a Trolox calibration curve. IC₅₀ values were not determined because serial sample concentrations were not tested.

### 2.8 ABTS radical scavenging activity

The ABTS radical-scavenging assay was performed according to Rufino et al. (17), with modifications based on Gupta et al. (18). The ABTS radical cation was generated by mixing 5 mL of ABTS stock solution with 88 μL of 140 mM potassium persulfate and incubating the mixture for 12 h in the dark at room temperature. Before analysis, the radical solution was diluted and analyzed at 734 nm. The reaction was initiated by mixing 30 μL of diluted sample with 3.0 mL of ABTS radical solution. Samples were analyzed without prior centrifugation. After 6 min, absorbance was measured at 734 nm using a PowerWave XS Microplate Spectrophotometer (BioTek Instruments, Inc., Winooski, United States). A Trolox calibration curve was prepared over the concentration range of 102.4–2,560 μM. Results were expressed as μM TE/mL. IC₅₀ values were not determined because serial sample concentrations were not tested.

### 2.9 Microbial community profiling

Microbial community composition was investigated by amplicon sequencing of the bacterial 16S rRNA gene and fungal internal transcribed spacer region. Total DNA was extracted from 500 mg of kombucha sample using the DNeasy PowerSoil DNA Kit (Qiagen, Hilden, Germany), following the manufacturer’s instructions. DNA concentration and purity were initially assessed using a NanoDrop spectrophotometer (Thermo Fisher Scientific, United States).

The bacterial 16S rRNA V3–V4 region and fungal ITS2 region were amplified using primer pairs 341F/805R and ITS3F/ITS4R, respectively (19, 20). All primers included Nextera overhang adapter sequences and are listed in Supplementary Supplementary Table S1.

Amplicons were quantified using the QuantiFluor/Quantus DNA quantification system (Promega, Madison, WI, United States) and sequenced using a P1 600-cycle kit on an Illumina NextSeq 1,000 platform in paired-end mode (2 × 300 bp).

Sequence data were processed using QIIME 2 version 2022.2 (21). Quality filtering, sequencing-error correction, chimera removal, and amplicon sequence variant inference were performed using DADA2 with default parameters (22). Bacterial taxonomy was assigned using a Naive Bayes classifier trained against GTDB release 207 (23), whereas fungal taxonomy was assigned against UNITE version 9.0 (24).

Relative abundances were calculated in R version 3.6.1 (25) using reshape2 version 1.4.3 (26), phyloseq version 1.14.0 (27), and vegan version 2.4.1 (28). Microbial community profiling was performed using eight samples: YMK-T0, YMK-T1, YMK-T2, YMK-T3, CHK-T0, CHK-T1, CHK-T2, and CHK-T3.

### 2.10 Statistical analysis

Chemical and antioxidant results are presented as mean ± standard deviation from three independent biological fermentations. Temporal differences were evaluated separately for each substrate using one-way analysis of variance followed by Tukey’s honestly significant difference test. Statistical significance was established at *p* < 0.05. Statistical analyses were performed in R version 3.6.1 using RStudio. Microbial relative-abundance data were analyzed descriptively because independent sequencing replicates were not available for all substrate–time combinations.

## 3 Results and discussion

### 3.1 Bioactive compounds and antioxidant capacity

The evolution of total phenolic content (TPC), total flavonoid content (TFC), and antioxidant activity throughout fermentation revealed clear substrate-dependent differences between yerba-mate kombucha (YMK) and coffee-husk kombucha (CHK) (Table 2). Overall, YMK consistently exhibited higher phenolic concentrations and stronger antioxidant capacity than CHK during the entire fermentation process, indicating that the initial phytochemical composition of the substrate strongly influenced the functional properties of the final beverage. YMK maintained higher TPC values than CHK at all sampling points, increasing from 356.16 ± 10.69 μg GAE/mL at T0 to 467.97 ± 43.09 μg GAE/mL at T4, whereas CHK fluctuated within a considerably narrower interval (188.15 ± 5.11–211.02 ± 37.07 μg GAE/mL). Similarly, TFC remained consistently higher in YMK, reaching a maximum value of 2.07 ± 0.09 μg RE/mL at T4, while CHK peaked at T3 (0.70 ± 0.01 μg RE/mL).

**Table 2:** Changes in total flavonoid content, total phenolic content, and antioxidant capacity of yerba-mate and coffee-husk kombuchas during fermentation and refrigerated storage.

Kombucha	Time	TFC (μg RE/mL)	TPC (μg GAE/mL)	ABTS (μM TE/mL)	DPPH inhibition (%)
YMK	T0	1.7 ± 0.04^bc^	356.16 ± 10.69^b^	1337.57 ± 101.87^ab^	73.31 ± 0.74^a^
YMK	T1	1.86 ± 0.14^b^	385.24 ± 15^b^	1783.43 ± 705.45^a^	71.51 ± 0.78^a^
YMK	T2	1.62 ± 0.10^c^	360.96 ± 23.05^b^	1631.19 ± 28.25^a^	72.4 ± 0.86^a^
YMK	T3	1.73 ± 0.02^bc^	377.05 ± 9.16^b^	728.6 ± 126.70^b^	72.17 ± 1.67^a^
YMK	T4	2.07 ± 0.09^a^	467.97 ± 43.09^a^	1506.13 ± 138.73^ab^	72.47 ± 0.51^a^
CHK	T0	0.35 ± 0.03^b^	210.17 ± 18.81^a^	218.58 ± 19.70^a^	41.82 ± 0.56^a^
CHK	T1	0.39 ± 0.07^b^	188.15 ± 5.11^a^	255.01 ± 35.13^a^	40.53 ± 0.64^a^
CHK	T2	0.34 ± 0.05^b^	199.44 ± 9.14^a^	267.76 ± 57.05^a^	43.76 ± 1.39^a^
CHK	T3	0.7 ± 0.01^a^	204.53 ± 9.92^a^	375.23 ± 116.73^a^	43.48 ± 2.13^a^
CHK	T4	0.35 ± 0.03^b^	211.02 ± 37.07^a^	302.37 ± 17.57^a^	42.96 ± 1.02^a^

Values are expressed as mean ± standard deviation from triplicate fermentations. T0: day 0; T1: day 3; T2: day 7; T3: day 9; T4: day 13, after refrigerated storage. YMK, yerba-mate kombucha; CHK, coffee-husk kombucha; TFC, total flavonoid content; TPC, total phenolic content. Different superscript letters within the same column for each respective kombucha type indicate statistically significant differences across timepoints (one-way ANOVA, Tukey HSD, *p* < 0.05).

The greater antioxidant-related responses observed for YMK are also consistent with the well-characterized phytochemical composition of *Ilex paraguariensis*. Previous studies have identified chlorogenic acids, particularly 5-caffeoylquinic acid and dicaffeoylquinic acid derivatives, together with caffeic acid derivatives, rutin, quercetin glycosides, and other flavonoids as the principal contributors to the antioxidant capacity of yerba-mate. During kombucha fermentation these compounds may undergo microbial transformation, hydrolysis, oxidation, or release from the plant matrix, thereby contributing to the antioxidant profile observed throughout fermentation. However, because individual phenolic compounds were not quantified in the present study, the contribution of specific metabolites should be regarded as literature-based rather than directly demonstrated (4, 8).

Although both substrates exhibited temporal oscillations in TFC, the magnitude of these fluctuations differed considerably, suggesting that microbial transformation of flavonoid precursors depended on the phytochemical composition of the starting material.

This oscillatory behavior has also been reported in alternative kombucha substrates, such as roselle-based analogues, and may reflect the concurrent release, transformation, and degradation of flavonoid compounds. Glycosylated flavonoids may be hydrolyzed by microbial β-glucosidases into aglycones, which can subsequently undergo oxidation, polymerization, or further microbial transformation (29).

ABTS antioxidant capacity showed an early peak in YMK at T1 (1783.43 ± 705.45 μM TE/mL) and a later maximum in CHK at T3 (375.23 ± 116.73 μM TE/mL). The higher ABTS values observed in YMK are consistent with its greater phenolic content throughout fermentation. Nevertheless, ABTS responses should not be interpreted as a direct proxy for TPC because individual phenolics, non-phenolic reductants, and compounds with different polarities contribute differently to the assay response (1, 30). Previous studies have similarly shown that the botanical matrix and fermentation conditions can markedly affect the antioxidant response of kombucha (4, 31).

In contrast to the ABTS response, DPPH inhibition showed no significant temporal variation within either formulation, remaining at approximately 71.51–73.31% in YMK and 40.53–43.76% in CHK. The consistently higher DPPH inhibition observed in YMK agrees with its greater phenolic and flavonoid contents. The different temporal patterns obtained with ABTS and DPPH may reflect differences in assay chemistry and in the relative contribution of individual antioxidant compounds. Therefore, the stable DPPH response should not be interpreted as an absence of chemical transformation during fermentation, but rather as a comparatively stable radical-scavenging response under the conditions of this assay. These results reinforce that the distinct phytochemical matrices of yerba-mate and coffee-husk shaped the biochemical trajectory of kombucha fermentation, influencing both the concentration of phenolic compounds and their antioxidant performance.

### 3.2 Organic acid dynamics and volatile metabolite evolution

#### 3.2.1 Organic acid dynamics

The organic acid profiles revealed distinct substrate- and time-dependent trajectories in YMK and CHK (Table 3). In kombucha fermentation, yeasts hydrolyze sucrose and convert the resulting monosaccharides into ethanol and other metabolites, which can subsequently be oxidized by acetic acid bacteria, including species of *Komagataeibacter* and *Acetobacter*, into organic acids (32, 33). Nevertheless, the concentrations observed in the present study did not follow a uniform or monotonic temporal pattern, indicating that the temporal profiles of the individual organic acids differed between substrates and across the fermentation stages.

**Table 3:** Organic acid concentrations in yerba-mate and coffee-husk kombuchas during fermentation and refrigerated storage.

Kombucha	Time point	Citric acid (mg/mL)	Acetic acid (mg/mL)	Malic acid (mg/mL)
YMK	T0	0.60 ± 0.00	2.02 ± 0.89	0.56 ± 0.00
YMK	T1	0.77 ± 0.06	2.46 ± 0.67	0.67 ± 0.03
YMK	T2	0.71 ± 0.05	1.93 ± 0.19	1.10 ± 0.34
YMK	T3	1.18 ± 0.08	2.24 ± 0.28	1.73 ± 0.30
YMK	T4	0.74 ± 0.11	2.16 ± 0.06	3.62 ± 0.40
CHK	T0	0.29 ± 0.01	1.48 ± 0.32	0.65 ± 0.11
CHK	T1	0.36 ± 0.01	1.06 ± 0.61	0.93 ± 0.34
CHK	T2	0.56 ± 0.27	NA	NA
CHK	T3	0.40 ± 0.13	NA	NA
CHK	T4	0.80 ± 0.06	1.89 ± 0.12	1.46 ± 0.06

Values are expressed as mean ± standard deviation from three independent biological fermentations. T0: day 0; T1: day 3; T2: day 7; T3: day 9; T4: day 13, after 4 days of refrigerated storage. NA indicates that the compound could not be reliably quantified because the chromatographic signal was below the analytical quantification limit or unsuitable for reliable integration.

The temporal evolution of organic acids is expected to depend on both the botanical substrate and the stage of fermentation because these factors influence nutrient availability, microbial succession, and metabolic activity. During kombucha fermentation, yeasts initially hydrolyze sucrose and produce ethanol and intermediate metabolites, whereas acetic acid bacteria subsequently oxidize ethanol and other substrates while simultaneously producing and consuming different organic acids. The chemical composition of the plant matrix further modulates these pathways by supplying distinct carbohydrates, phenolic compounds, minerals, and organic-acid precursors, resulting in substrate-dependent fermentation trajectories (3, 4, 34, 35).

Citric, acetic, and malic acids were quantitatively reported in both formulations, whereas lactic, butyric, and isovaleric acids were detected inconsistently or could not be reliably quantified. In YMK, acetic acid was present at 2.02 ± 0.89 mg/mL at T0 and reached its highest concentration at T1, at 2.46 ± 0.67 mg/mL. It subsequently decreased to 1.93 ± 0.19 mg/mL at T2, increased to 2.24 ± 0.28 mg/mL at T3, and reached 2.16 ± 0.06 mg/mL at T4. Thus, acetic acid exhibited temporal fluctuations rather than continuous accumulation. CHK presented lower acetic acid concentrations at the time points for which quantitative data were available, decreasing from 1.48 ± 0.32 mg/mL at T0 to 1.06 ± 0.61 mg/mL at T1 and reaching 1.89 mg/mL at T4.

The acetic acid concentrations detected in both formulations were within or below ranges previously reported for traditional black- and green-tea kombuchas, for which values of approximately 3.06 and 3.76 mg/mL, respectively, have been described (7). Moderate acetic acid concentrations have been associated with a less pronounced vinegar-like character, whereas excessive volatile acidity may negatively affect sensory acceptance (7). However, because sensory analysis was not performed in the present study, the effect of the measured concentrations on consumer perception cannot be directly established.

The temporal fluctuations observed for acetic acid most likely reflect the dynamic equilibrium between ethanol production by fermentative yeasts and its subsequent oxidation by acetic acid bacteria. Rather than accumulating continuously, acetic acid concentrations may vary according to changes in microbial activity, substrate availability, oxygen-dependent oxidation processes, and the progressive depletion of fermentable carbohydrates (34). Differences between YMK and CHK are also likely influenced by their contrasting phytochemical composition and nutrient accessibility, which may modulate the relative activities of yeasts and acetic acid bacteria throughout fermentation. Consequently, the observed acetic-acid profiles probably represent coordinated microbial and metabolic responses rather than a simple linear accumulation process (3, 35).

Malic acid showed the most pronounced temporal change in YMK, increasing from 0.56 ± 0.00 mg/mL at T0 to 0.67 ± 0.03 mg/mL at T1, 1.10 ± 0.34 mg/mL at T2, and 1.73 ± 0.30 mg/mL at T3, before reaching 3.62 ± 0.40 mg/mL at T4. In CHK, malic acid increased from 0.65 ± 0.11 mg/mL at T0 to 0.93 ± 0.34 mg/mL at T1 and reached 1.46 ± 0.06 mg/mL at T4. The final concentration was therefore considerably higher in YMK than in CHK. Although malic acid transformation can be influenced by the metabolic capacities of the microbial consortium, the present data do not allow the involvement or absence of malolactic activity to be demonstrated (7).

Citric acid was detected from T0 onward in both formulations. In YMK, its concentration increased from 0.60 ± 0.00 mg/mL at T0 to a maximum of 1.18 ± 0.08 mg/mL at T3, followed by a decrease to 0.74 mg/mL at T4. In CHK, citric acid increased from 0.29 ± 0.01 mg/mL at T0 to 0.80 mg/mL at T4, with intermediate fluctuations. Citric acid may originate from the plant substrate or from microbial metabolic transformations during fermentation; however, the present concentration data alone cannot distinguish between extraction, preservation, consumption, and *de novo* production through central carbon metabolism (33).

The different trajectories of acetic, malic, and citric acids indicate that the two botanical matrices supported distinct fermentation and storage responses. Differences in the native composition and accessibility of substrates may have influenced organic-acid metabolism and preservation during the process (7, 31, 33). However, the concentration profiles alone do not permit individual precursor–product pathways to be assigned.

Butyric, isovaleric, and lactic acids produced weak or inconsistently quantifiable chromatographic signals and were therefore not subjected to temporal interpretation. Only citric, acetic, and malic acids are quantitatively discussed.

Collectively, the organic-acid profiles demonstrate that YMK and CHK followed distinct chemical trajectories. YMK was particularly characterized by the increase in malic acid, whereas acetic and citric acids exhibited non-monotonic variation in both formulations. The changes observed at T4 should be interpreted in the context of refrigerated storage rather than as a direct continuation of active fermentation.

#### 3.2.2 Volatile metabolite evolution

**Figure 1:**
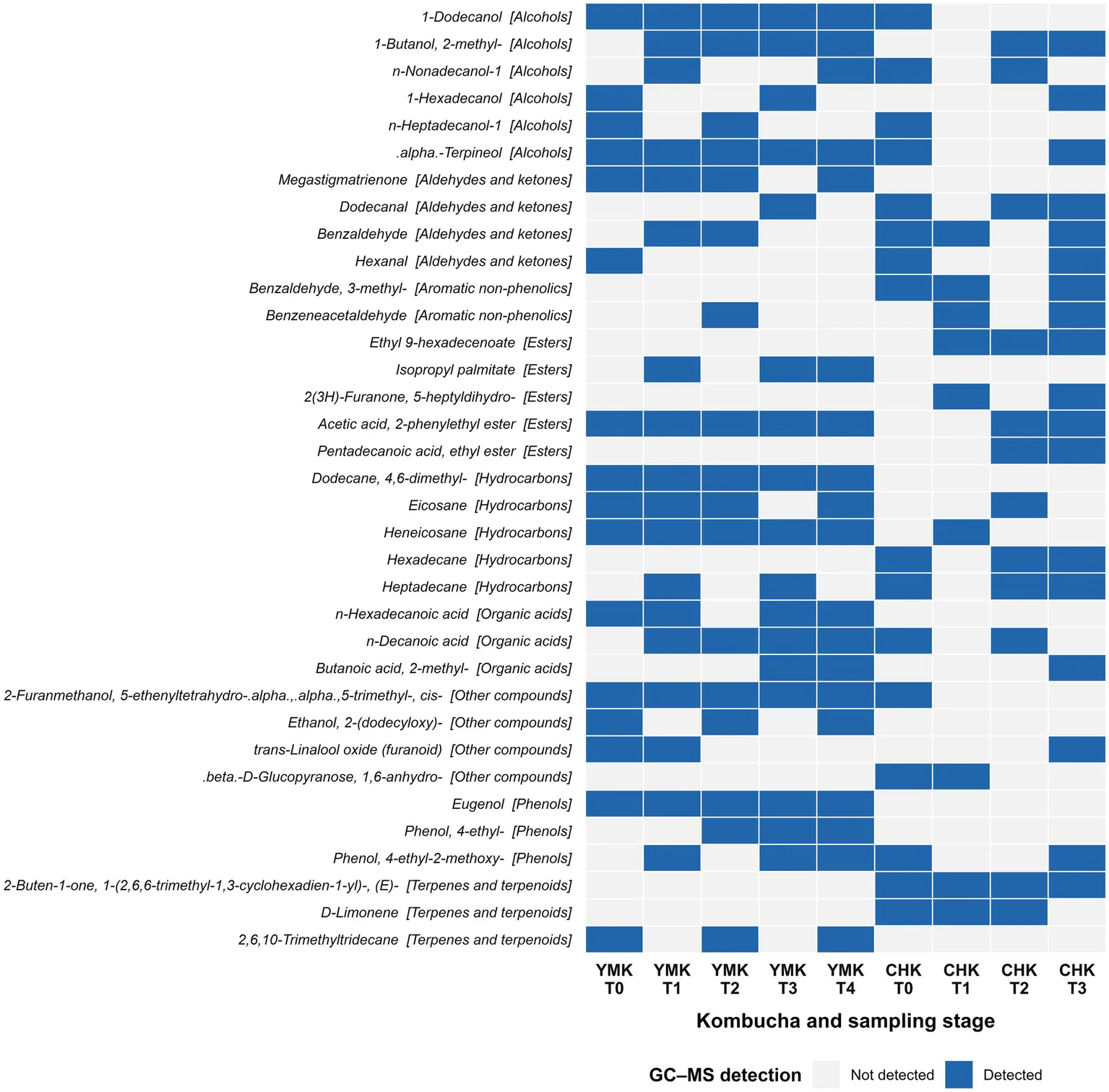
Temporal detection patterns of selected informative volatile and semi-volatile compounds in yerba-mate and coffee-husk kombuchas. Blue cells indicate detection by GC–MS, whereas gray cells indicate no detection. Compounds were selected to represent persistent, transient, substrate-associated, or fermentation-associated patterns across the evaluated sampling stages. Signals provisionally classified as likely analytical artifacts were excluded. YMK was analyzed from T0 to T4, whereas CHK was analyzed from T0 to T3 because the corresponding T4 sample was unavailable for GC–MS analysis. YMK, yerba-mate kombucha; CHK, coffee-husk kombucha; T0: Day 0; T1: Day 3; T2: Day 7; T3: Day 9; T4: Day 13, after refrigerated storage.

**Figure 2:**
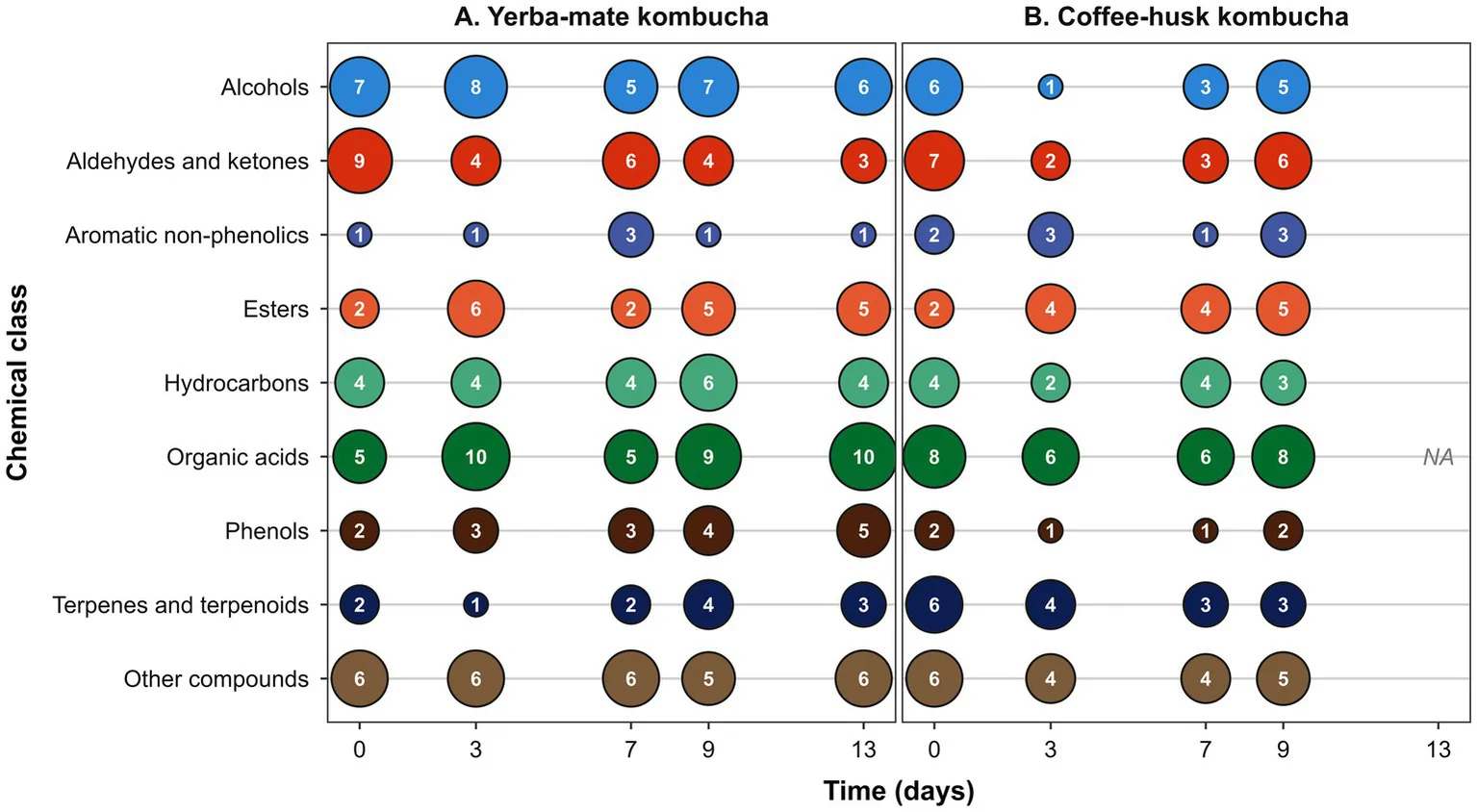
Temporal distribution of volatile chemical classes in **(A)** yerba-mate kombucha and **(B)** coffee-husk kombucha. Each point represents the number of uniquely detected non-artifact compounds, based on unique InChIKey identifiers, within each chemical class and sampling stage. T0: day 0; T1: day 3; T2: day 7; T3: day 9; T4: day 13, after 4 days of refrigerated storage. GC–MS data were not available for coffee-husk kombucha at T4; therefore, this time point is indicated as not analyzed rather than assigned a value of zero.

GC–MS profiling revealed temporal changes in the volatile and semi-volatile composition of both formulations during fermentation and, for YMK, during refrigerated storage (Figures 1, 2). Following deduplication using unique InChIKey identifiers and exclusion of signals provisionally classified as likely analytical artifacts, the curated dataset contained 91 putatively identified compounds. Of these, 74 were detected in at least one YMK sample and 60 in at least one CHK sample; 43 were shared between formulations, whereas 31 were detected only in YMK and 17 only in CHK. Representative compounds displaying persistent, recurrent, substrate-associated, or potentially artifactual detection patterns are summarized in Table 1. Temporal detection patterns for selected informative compounds are shown in Figure 1, the temporal distribution of chemical classes is presented in Figure 2, and shared and condition-specific intersections within the selected compound subset are summarized in Supplementary Supplementary Figure S1.

The two substrates exhibited different temporal distributions of chemical classes (Figure 2). YMK maintained a relatively broad profile throughout fermentation and refrigerated storage, with between 36 and 45 unique non-artifact compounds detected at individual sampling points. CHK exhibited between 27 and 43 compounds from T0 to T3. VOC data were not available for CHK at T4, and this time point should therefore be reported as not analyzed rather than interpreted as having no detected compounds.

Alcohols, aldehydes and ketones, esters, hydrocarbons, organic acids, phenols, terpenes and terpenoids, and aromatic non-phenolic compounds were detected in both matrices. The occurrence of these classes is consistent with the chemical complexity of the original plant materials and with microbial transformations occurring during kombucha fermentation. Yeast metabolism can generate ethanol, higher alcohols, aldehydes, and other aroma-active metabolites, while acetic acid bacteria can oxidize ethanol and aldehydes into their corresponding acids (5, 33).

Phenylethyl alcohol and selected branched-chain alcohols were detected across multiple fermentation stages in both formulations. Such higher alcohols may be generated through sugar metabolism or amino acid catabolism, including reactions commonly associated with the Ehrlich pathway (36). Alcohols can also participate in ester formation through reactions involving organic acids, contributing to fruity and floral sensory characteristics in fermented beverages (31, 37). Nevertheless, precursor concentrations, enzyme activities, and esterification rates were not measured here; these pathways should therefore be considered plausible interpretations rather than demonstrated mechanisms.

Several esters, including acetate and fatty-acid-derived compounds, occurred intermittently during fermentation. Ester production in kombucha may contribute to the attenuation of sharp acidic notes by introducing sweet, fruity, or floral aromas (33). However, the qualitative presence/absence approach does not demonstrate that ester concentrations increased during fermentation, nor does it establish their actual sensory relevance.

Terpenes and terpenoid-related compounds also contributed to the profiles of both substrates. Linalool was detected repeatedly in YMK and CHK, while α-terpineol, geraniol, carvone-related compounds, and other terpenoids displayed more substrate- or time-specific patterns. These compounds may be directly extracted from yerba-mate and coffee-husk or generated through the transformation of native aroma precursors (31, 38). Their presence may contribute floral, citrus, herbal, or rose-like notes. Nevertheless, the statement that these terpenes were synthesized or consumed by the microbiota cannot be established from detection data alone. Microbial transformation remains a plausible explanation for their temporal variation, particularly because terpene persistence in fermented matrices can depend on both substrate chemistry and microbial metabolism (31).

YMK showed recurrent detection of linalool, α-terpineol, eugenol, megastigmatrienone, phenylethyl alcohol, and several medium-chain organic acids. The broader distribution of terpenes, phenols, and aromatic compounds in YMK is consistent with the chemically complex composition of *Ilex paraguariensis*. In contrast, CHK exhibited a distinct combination of aldehydes, esters, hydrocarbons, and terpenoid-related compounds, reflecting the composition and prior processing of coffee-husk.

Aldehydes and ketones exhibited temporal changes in both formulations. Nonanal and decanal were detected across several time points and may contribute citrus-like, waxy, green, or fatty sensory notes. Hexanal was detected intermittently and may be associated with green or grassy characteristics. Aldehydes can serve as intermediate metabolites that are either reduced to alcohols through alcohol dehydrogenase-associated reactions or oxidized to carboxylic acids through aldehyde dehydrogenase-associated reactions (3, 35).

Furfural was detected during the early stages of CHK. Because furfural can arise from carbohydrate degradation during drying or other thermal processing, its occurrence may partly reflect the previous processing of the coffee-derived substrate rather than fermentation alone (9, 37).

Organic acids detected in the volatile profile included branched-chain, medium-chain, and long-chain acids. Compounds such as 3-methylbutanoic, octanoic, dodecanoic, and pentadecanoic acids were detected at multiple stages. Some of these molecules may originate from microbial lipid or amino acid metabolism and can contribute sour, fatty, rancid, or cheese-like sensory notes depending on concentration (31). Medium-chain fatty acids have also been reported to exhibit antimicrobial activity, but the qualitative detection of these compounds in the present study does not demonstrate that they occurred at biologically effective concentrations (31).

Phenolic and alkaloid-related signals were also detected. Caffeine remained detectable across the monitored stages, consistent with its origin in the plant matrices and its relative persistence during fermentation. Caffeine is nutritionally relevant because of its stimulant activity and contribution to bitterness, although the present GC–MS analysis did not quantify its concentration or dietary exposure (9). Therefore, caffeine should be described as qualitatively detected rather than as a principal functional component of the beverages.

The repeated detection of 2,4-di-tert-butylphenol requires particular caution. Although this compound has been described as possessing biological activity, it may also originate from plastics, laboratory materials, environmental contamination, or analytical procedures. It should therefore not be presented as a principal antioxidant constituent without confirmation using an authentic standard and appropriate blank controls. The same caution applies to phthalate derivatives, silanediol-related compounds, homosalate, halogenated hydrocarbons, and plasticizer-like esters. These signals were retained in Table 1 for transparency but excluded from the biological and nutritional interpretation.

Accordingly, the VOC dataset was interpreted qualitatively as a description of compound detection and chemical diversity. Confirmation of the principal substrate- and fermentation-associated markers will require blank-controlled analysis, retention-index verification, authentic standards, and relative or absolute quantification.

Across the available sampling stages, YMK and CHK exhibited distinct qualitative volatile profiles. YMK showed a broader and more persistent distribution of detected chemical classes, whereas CHK displayed a more variable profile containing several coffee-husk-associated aldehydes, esters, and terpenoids. Because many of the detected compounds may result from or be modified by yeast and acetic acid bacterial metabolism, these chemical patterns provide an appropriate basis for examining the bacterial and fungal community profiles described in Section 3.3 (5, 33).

Because the present study focused on chemical profiling, the detected volatile compounds should not be interpreted as direct contributors to aroma perception. Future studies combining GC–MS with GC-olfactometry, electronic-nose analysis, and sensory evaluation would enable identification of aroma-active compounds and establish direct relationships between volatile composition and sensory attributes.

### 3.3 Microbial community structure and succession during fermentation

**Figure 3:**
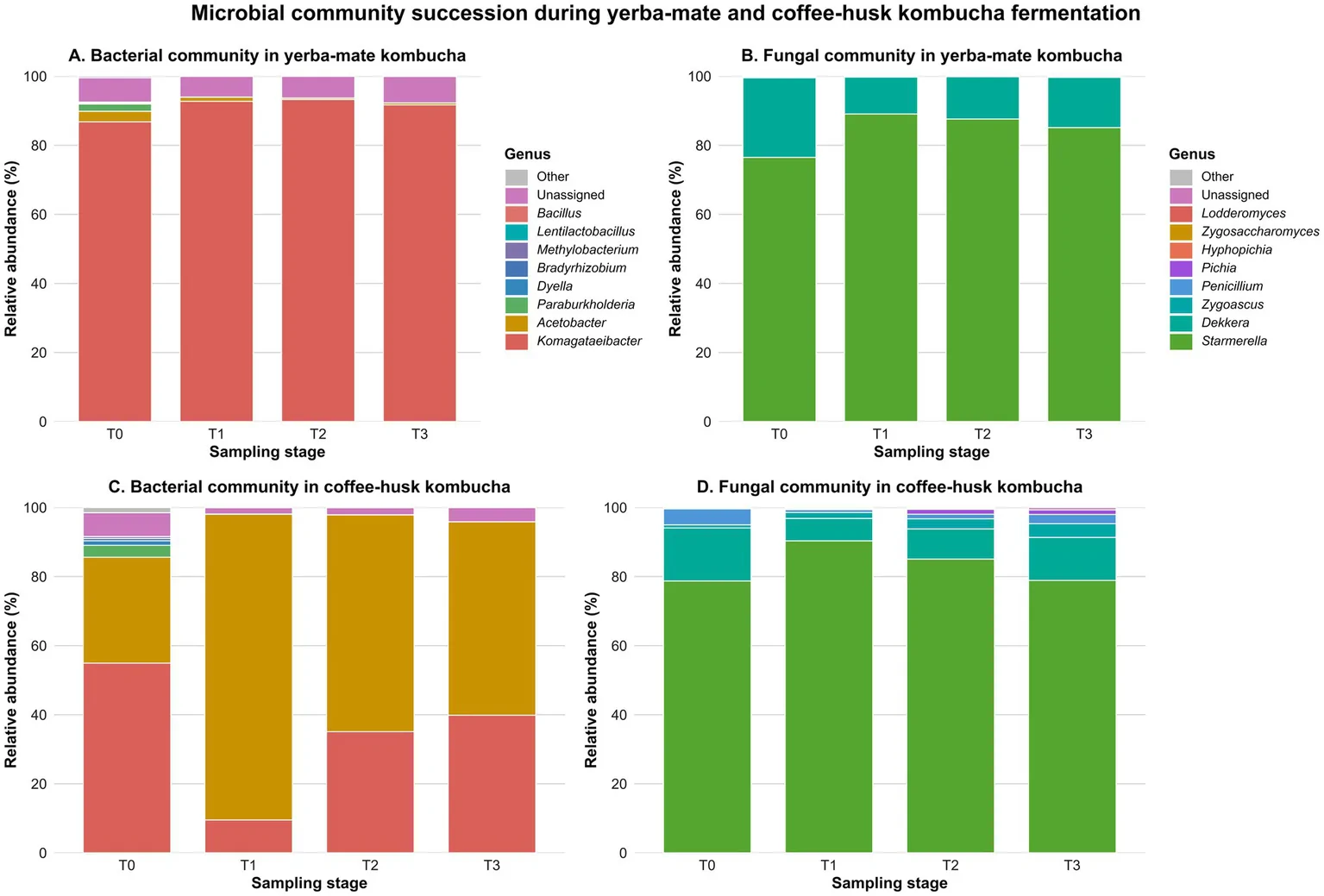
Bacterial and fungal community succession during yerba-mate and coffee-husk kombucha fermentation. **(A)** Bacterial community in yerba-mate kombucha. **(B)** Fungal community in yerba-mate kombucha. **(C)** Bacterial community in coffee-husk kombucha. **(D)** Fungal community in coffee-husk kombucha. Relative abundance was determined using bacterial 16S rRNA gene and fungal ITS2 amplicon sequencing. Taxa not assigned at the genus level are shown as “Unassigned,” whereas low-abundance taxa are grouped as “Other.” T0–T3 indicate the analyzed sampling stages.

Amplicon sequencing revealed substrate-associated differences in the bacterial communities of yerba-mate kombucha (YMK) and coffee-husk kombucha (CHK), whereas the fungal communities remained comparatively stable throughout the analyzed stages (Figure 3). Hierarchical taxonomic assignments from phylum to species level are presented in Supplementary Supplementary Figure S2. Because bacterial 16S rRNA and fungal ITS2 libraries were generated independently, relative abundances should be interpreted within each microbial domain and not as direct quantitative comparisons between bacteria and fungi.

The bacterial community of YMK was consistently dominated by *Komagataeibacter*, with smaller contributions from *Acetobacter* and other low-abundance genera. This persistence indicates that the yerba-mate matrix supported a comparatively stable acetic acid bacterial structure. In contrast, CHK showed a more pronounced temporal shift between *Komagataeibacter* and *Acetobacter*. *Komagataeibacter* contributed substantially at the initial stage, whereas *Acetobacter* became the predominant bacterial genus at T1 and remained an important component at subsequent stages. These descriptive patterns are consistent with substrate-associated differences in the balance between the principal acetic acid bacterial groups.

*Komagataeibacter* was already the dominant bacterial genus at T0, indicating that its predominance was established immediately after inoculation rather than arising during fermentation. Because the parental SCOBY was not sequenced independently, the present study cannot distinguish the relative contributions of the inoculum and the native microbiota associated with the botanical substrates. Nevertheless, the persistence of *Komagataeibacter* throughout fermentation is consistent with previous reports describing this genus as a dominant member of mature kombucha consortia due to its high acid tolerance and cellulose-producing capacity (3, 32, 39).

Fungal profiles were dominated by *Starmerella* in both formulations and at all evaluated stages. *Starmerella* was already dominant at T0, suggesting that it was primarily introduced with the starter culture rather than selectively emerging during fermentation. Because the parental SCOBY was not analyzed independently, the relative contributions of the inoculum and substrate-associated microbiota cannot be fully separated. Nevertheless, its persistence in both formulations indicates that this yeast was well adapted to the sugar-rich and progressively acidic fermentation environment (35, 39, 40). *Dekkera* represented the principal secondary genus, while *Zygoascus*, *Zygosaccharomyces*, *Pichia*, *Hyphopichia*, and other yeasts occurred at lower relative abundances. The persistence of *Starmerella* and *Dekkera* suggests that the dominant fungal component of the parental inoculum was comparatively robust to the chemical differences between yerba-mate and coffee-husk.

The stronger bacterial reorganization observed in CHK coincided with the distinct organic-acid and volatile-compound patterns described in Section 3.2. Acetic acid bacteria can transform ethanol, aldehydes, and other microbial metabolites, while yeasts contribute alcohols and aroma precursors. Nevertheless, the current study did not test statistical associations between individual taxa and chemical compounds. Therefore, the parallel temporal patterns should be interpreted as substrate-associated observations rather than direct evidence of taxon–metabolite causality.

Several low-abundance bacterial and fungal genera were also detected, including *Bradyrhizobium*, *Methylobacterium*, *Paraburkholderia*, *Pichia*, *Hyphopichia*, and *Penicillium*. These taxa may have originated from the botanical materials, the SCOBY inoculum, or the processing environment. Their precise origin and metabolic contribution cannot be determined from relative-abundance data alone. Studies of coffee-associated fermentations have similarly reported diverse yeasts and bacteria derived from the raw material and processing environment (37, 41).

Detection of low-abundance filamentous fungi by amplicon sequencing does not demonstrate viability, growth, toxin production, or product spoilage. Such signals may represent DNA derived from the raw substrate or environmental background. Consequently, their biological relevance would require confirmation by culture-dependent methods or targeted analysis.

Overall, both botanical matrices maintained the principal microbial groups associated with kombucha fermentation but promoted different bacterial trajectories. YMK was characterized by the persistent dominance of *Komagataeibacter*, whereas CHK showed a stronger temporal contribution from *Acetobacter*. In contrast, the fungal communities were more stable and remained dominated by *Starmerella* and *Dekkera*. These findings support the conclusion that substrate composition modulated microbial succession without displacing the core acetic acid bacteria and fermentative yeasts of the kombucha consortium.

Future studies combining microbiome sequencing with correlation network analyses or other multivariate statistical approaches may further elucidate microorganism–metabolite relationships during kombucha fermentation.

## 4 Conclusion

Yerba-mate and coffee-husk infusions supported distinct kombucha fermentation trajectories, generating beverages with substrate-dependent chemical and microbial profiles. YMK consistently exhibited higher phenolic and flavonoid contents and stronger antioxidant responses, whereas CHK displayed lower but comparatively stable antioxidant-related measurements. Organic-acid and volatile-compound patterns also differed between formulations, with YMK showing a higher final malic acid concentration.

Both formulations retained a core kombucha community composed predominantly of acetic acid bacteria and fermentative yeasts. YMK was characterized by persistent bacterial dominance of *Komagataeibacter*, whereas CHK showed a stronger temporal contribution from *Acetobacter*. The fungal communities were more stable and were dominated by *Starmerella* and *Dekkera*. Overall, the results demonstrate that substrate composition modulated microbial succession and metabolite profiles while preserving the principal functional groups of the kombucha consortium. Yerba-mate provided the highest antioxidant-related chemical profile under the evaluated conditions, while coffee-husk showed potential as a value-added substrate for the development of differentiated fermented beverages and the valorization of coffee-processing residues.

Future studies should include a conventional *Camellia sinensis*-based kombucha fermented under identical conditions to directly benchmark these alternative substrates against traditional kombucha and further elucidate substrate-specific effects on microbial succession and metabolite production.

## Data Availability

The data analyzed in this study are available in the NCBI SRA under accession number PRJNA1521551: https://www.ncbi.nlm.nih.gov/sra/PRJNA1521551.
