# Supplementary material for: Fermentation dynamics of yerba-mate and coffee-husk kombuchas: bioactive compounds, organic acids, volatile metabolites, and microbial succession
Source: Front Nutr. 2026 Sep 3;13:1935769. doi: 10.3389/fnut.2026.1935769 (PMC13584766; doi:10.3389/fnut.2026.1935769)
Supplement: Supplementary file 1 [file Data_Sheet_1.PDF]

Supplementary Table S1. Primers and sequencing conditions used for bacterial and fungal community profiling.

Target	Primer	Sequence (5′–3′)	Sequencing platform
**ITS2 (Fungi)**	ITS_3F	TCGTCGGCAGCGTCAGATGTGTATAAGA GACAGGCATCGATGAAGAACGCAGC	Illumina NextSeq 1000 (2 × 300 bp)
**ITS2 (Fungi)**	ITS_4R	GTCTCGTGGGCTCGGAGATGTGTATAAGA GACAGTCCTCCGCTTATTGATATGC	Illumina NextSeq 1000 (2 × 300 bp)
**16S rRNA (V3–V4)**	341F	TCGTCGGCAGCGTCAGATGTGTATAAGA GACAGCCTACGGGNGGCWGCAG	Illumina NextSeq 1000 (2 × 300 bp)
**16S rRNA (V3–V4)**	805R	GTCTCGTGGGCTCGGAGATGTGTATAAGA GACAGGACTACHVGGGTATCTAATCC	Illumina NextSeq 1000 (2 × 300 bp)

All primers included Illumina Nextera adapter overhang sequences according to the manufacturer's protocol. DNA was extracted from 500 mg of kombucha using the DNeasy PowerSoil DNA Kit (Qiagen). Amplicons were quantified using the Quantus Fluorometer (Promega) and sequenced on an Illumina NextSeq 1000 platform using the P1-600 kit (paired-end, 2 × 300 bp). Sequence processing was performed with QIIME2 v2022.2 using DADA2 for denoising and ASV inference. Taxonomic assignment was conducted using a Naïve Bayes classifier with GTDB v207 for bacteria and UNITE v9.0 for fungi.

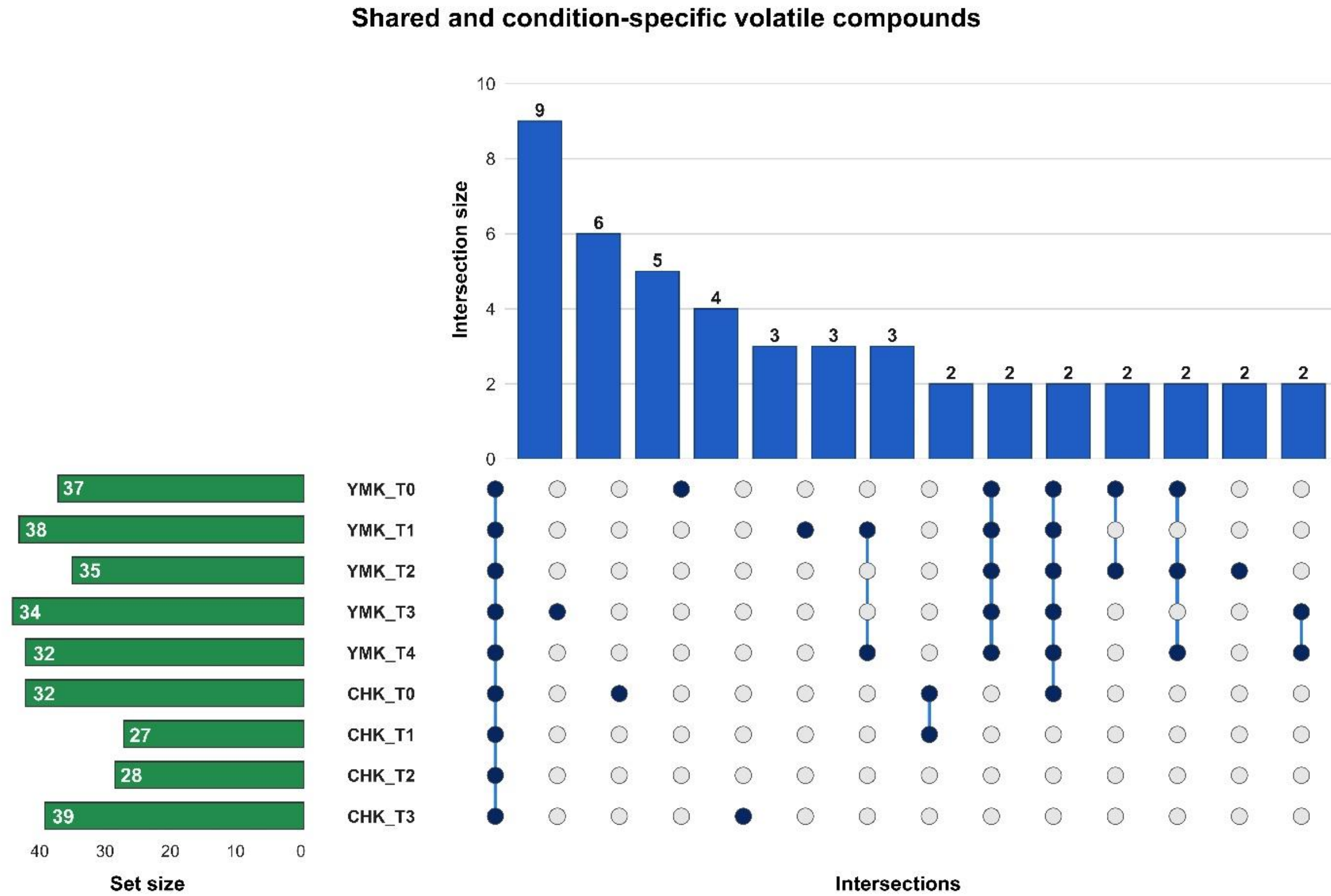

Supplementary Figure S1. Shared and condition-specific patterns within the selected subset of informative volatile compounds. Vertical bars represent intersection sizes, horizontal bars represent the number of selected informative compounds detected in each sample, and connected dots indicate the samples contributing to each intersection. The plot does not represent the complete curated GC–MS dataset. YMK: yerba-mate kombucha; CHK: coffee-husk kombucha.

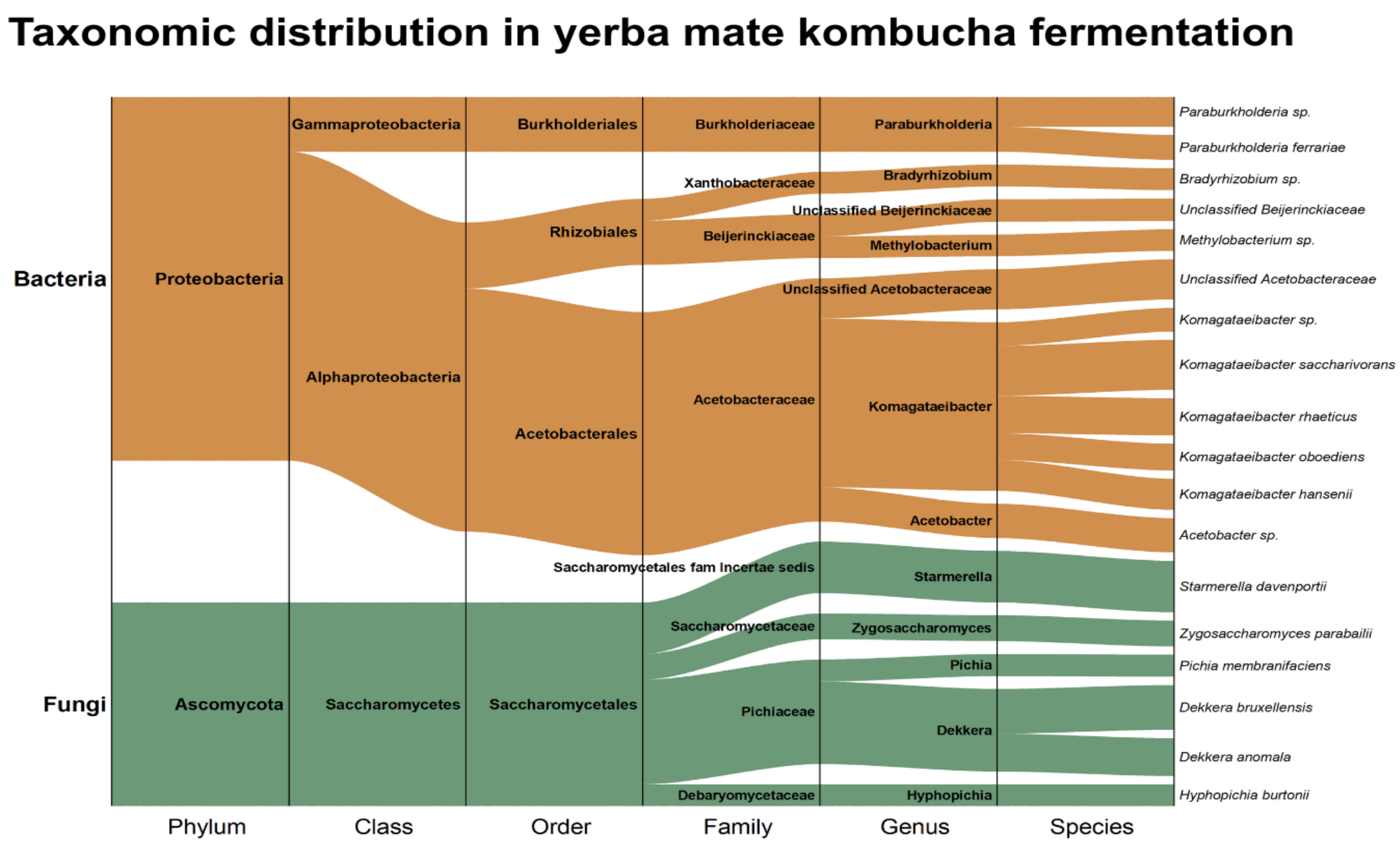

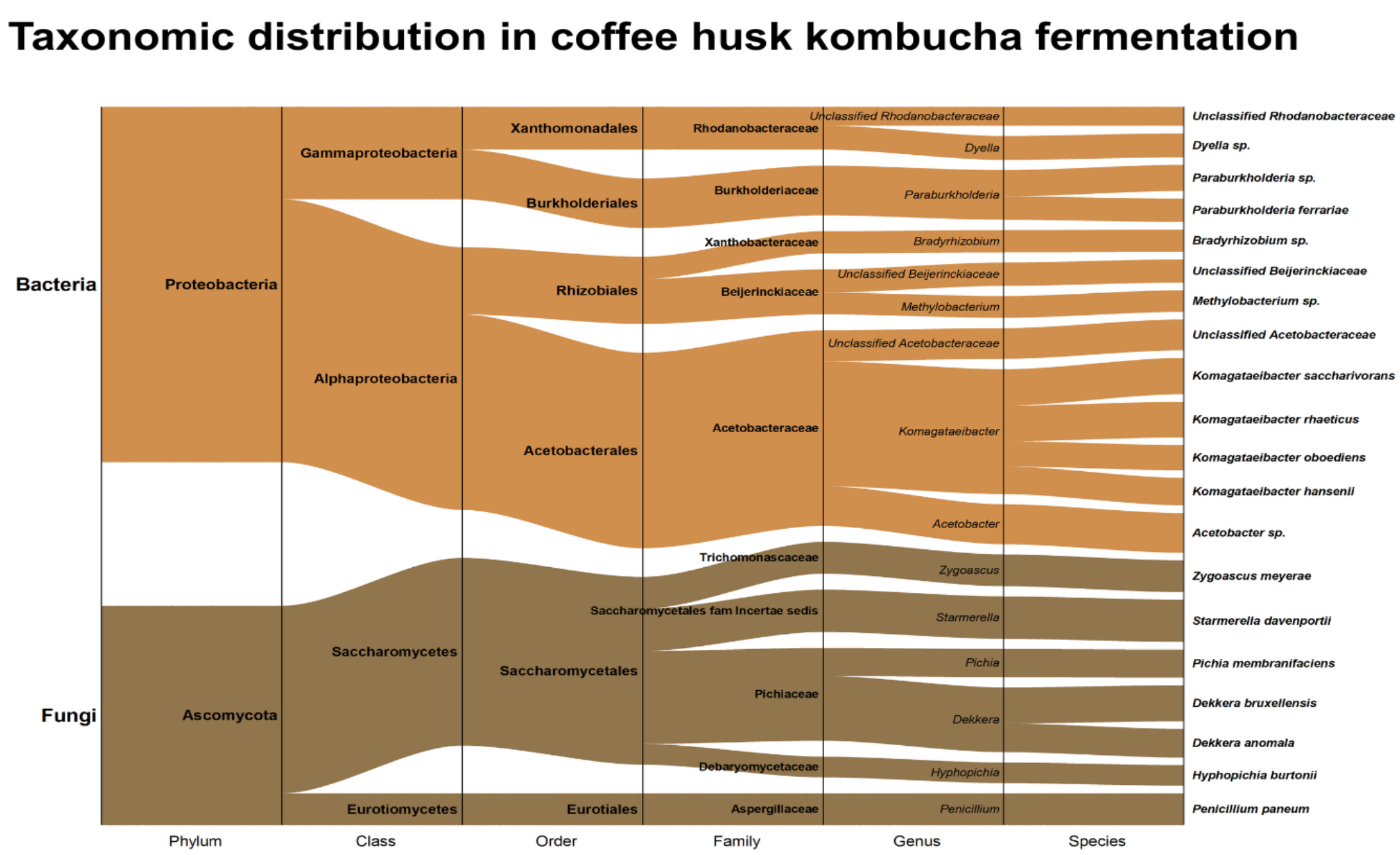

Supplementary Figure S2. Hierarchical taxonomic distribution of bacterial and fungal communities in yerba-mate and coffee-husk kombuchas. Alluvial diagrams show taxonomic assignments from phylum to species level for (A) yerba-mate kombucha and (B) coffee-husk kombucha. Flow widths represent relative abundance within the bacterial or fungal dataset; bacterial and fungal flow widths should not be interpreted as direct quantitative comparisons between 16S and ITS libraries.
